# Natural polysaccharides as immune adjuvants to overcome immune checkpoint inhibitor resistance: mechanistic insights, clinical evidence, and translational challenges

**DOI:** 10.3389/fimmu.2026.1914914

**Published:** 2026-08-06

**Authors:** Yuhua Li, Jiao Qian, Shurong Zhou, Chunyan Wang, De Cai

**Affiliations:** 1 Department of Pharmacy, Affiliated Hospital of Guangdong Medical University, Zhanjiang, Guangdong, China; 2 Department of Pharmacy, The First Naval Hospital of Southern Theatre Command, Zhanjiang, Guangdong, China; 3 Department of Pharmacy, Changhai Hospital, Naval Military Medical University, Shanghai, China; 4 Department of General Surgery, The First Naval Hospital of Southern Theatre Command, Zhanjiang, Guangdong, China; 5 Department of Pharmacy, No. 971 Hospital of the People’s Liberation Army Navy, Qingdao, Shandong, China

**Keywords:** biomanufacturing, clinical translation, glycomics, gut microbiota, immune adjuvants, immune checkpoint inhibitors, natural polysaccharides

## Abstract

Immune checkpoint inhibitors (ICIs) have redefined the treatment landscape of advanced malignancies, yet primary and acquired resistance severely limits their therapeutic efficacy across most solid tumors. Even in biomarker-stratified patient cohorts, objective response rates frequently fall below 25%. Natural polysaccharides have emerged as a unique category of immune adjuvants. Beyond activating innate immune receptors including Toll-like receptor 4 and downstream MyD88 signaling, such macromolecules remodel the gut microbiota–metabolite axis and partially reverse immunosuppressive tumor microenvironments. Cumulative preclinical findings and early-phase clinical observations suggest synergistic therapeutic benefits for polysaccharide plus ICI combinations. Still, robust evidence from large-scale randomized Phase III trials remains absent, and polysaccharides cannot yet be validated as standard adjuvants to resolve ICI resistance. In this review, we systematically dissect the structural basis governing polysaccharide immunomodulation and elaborate four core mechanisms for overcoming immunotherapy resistance: repolarizing tumor-associated macrophages toward anti-tumor phenotypes, alleviating CD8^+^ T cell exhaustion, regulating bidirectional gut microbiota-immune crosstalk, and inhibiting tumor-intrinsic immune escape cascades. We further summarize persistent translational bottlenecks: structural heterogeneity, nonstandard quality control, low oral bioavailability, and inconsistent clinical trial protocols, and conduct a head-to-head comparison between polysaccharides and mainstream immunostimulants (CpG oligodeoxynucleotides, STING agonists, fecal microbiota transplantation). Advanced technical tools including AI-assisted glycomics and graph neural networks, alongside synthetic biology platforms, provide new solutions to resolve structural identification and mass-production limitations. Integrating polysaccharide production with Industry 6.0 intelligent manufacturing and circular bioeconomy models can balance therapeutic development and global sustainable development targets. Finally, we propose a precision medicine workflow incorporating multi-layer biomarkers (gut microbial profiles, intratumoral immune signatures, glycomic fingerprints) to optimize patient stratification and individualized dosing. Rather than presenting rigid standardized treatment schemes, this review constructs a realistic, mechanism-guided roadmap to accelerate the clinical translation of natural polysaccharide ICI adjuvants.

## 1 Introduction

### 1.1 Clinical application status and universal resistance of ICIs

Immune checkpoint inhibitors (ICIs), predominantly anti-PD-1/PD-L1 monoclonal antibodies, have reshaped the therapeutic landscape of advanced solid tumors over the past decade by re-invigorating exhausted host T cells. They have been approved as first-line or subsequent therapies for non-small cell lung cancer (NSCLC), hepatocellular carcinoma, melanoma, and colorectal cancer, and are now standard of care for multiple advanced malignancies (1). However, accumulating real-world data reveal that primary and acquired resistance remain the principal barriers to durable clinical benefit. A substantial proportion of patients fail to achieve lasting responses, and even in biomarker-selected populations single-agent ICI activity is frequently modest (1). These limitations underscore that ICI monotherapy cannot overcome the widespread challenge of drug resistance.

The adverse event profile of ICIs further constrains their utility. Postow et al. systematically reviewed immune-related adverse events and established that these toxicities span virtually every organ system, with severe (grade 3–4) events occurring in 10–15% of patients and treatment-related deaths reported across major ICI trials (1). This safety burden necessitates either treatment interruption or permanent discontinuation in a substantial proportion of patients, limiting the feasibility of long-term ICI administration.

Current salvage strategies—targeted small-molecule inhibitors and CAR-T cell therapy—each carry intrinsic limitations. Sterner RC et al. delineated the barriers to effective CAR-T therapy: severe life-threatening toxicities (cytokine release syndrome, immune effector cell-associated neurotoxicity), modest anti-tumor activity in solid tumors, antigen escape, restricted trafficking, and limited tumor infiltration (2). They further noted that the host-tumor microenvironment (TME) interplay critically alters CAR-T function, such that even patients who respond initially often relapse due to TME-mediated suppression. Targeted kinase inhibitors fare no better: most are developed against single genetic alterations, and secondary mutations precipitate rapid treatment failure, accompanied by hepatorenal toxicity and severe cutaneous reactions (2).

Against this backdrop, natural polysaccharides derived from medicinal plants, edible fungi, and marine organisms have emerged as promising adjuvant candidates. Unlike synthetic small molecules, these macromolecules are characterized by low systemic toxicity, multi-pathway modulation of the TME, and bidirectional regulation of gut microbiota homeostasis (3). Huang J et al. provided the first causal evidence linking polysaccharide administration to ICI sensitization *in vivo*: ginseng polysaccharides (GPs) combined with anti-PD-1/PD-L1 monoclonal antibodies increased anti-tumor response by remodeling gut microbiota composition, elevating the microbial metabolite valeric acid, and reducing the kynurenine/tryptophan (Kyn/Trp) ratio—a surrogate of IDO-mediated immunosuppression (3). Responders exhibited enriched *Parabacteroides distasonis* and *Bacteroides vulgatus*, and fecal microbiota transplantation (FMT) from non-responders to germ-free mice restored PD-1 inhibitor sensitivity, establishing that the adjuvant effect of polysaccharides is mechanistically dependent on the gut microbiome (3).

### 1.2 Limitations of existing review articles

Existing reviews on polysaccharides and cancer immunotherapy are fragmented in scope and fail to bridge structural biology with translational evidence. Wang XY et al. compiled the structural determinants of anti-breast-cancer polysaccharides but explicitly acknowledged that most structure-activity relationship studies remain confined to *in vitro* or animal experiments, with a paucity of clinical translation data (4). This gap between bench and bedside is precisely where the present review is positioned.

A second critical gap concerns PD-L1 glycosylation—a post-translational modification that directly interferes with patient stratification for checkpoint blockade. Li CW et al. demonstrated that N-glycosylation at N192, N200, and N219 antagonizes GSK3β binding to PD-L1, thereby preventing β-TrCP-mediated recruitment of the E3 ubiquitin ligase complex and subsequent proteasomal degradation (5). By stabilizing PD-L1 on the tumor cell surface, this glycosylation event suppresses T-cell activity and facilitates immune evasion. Lee HH et al. extended this finding clinically: because N-linked glycosylation accounts for approximately 52% (17 kDa of a predicted 33 kDa) of observed PD-L1 molecular weight, conventional immunohistochemical detection yields false-negative results in 10-20% of patients (6). Their enzymatic deglycosylation protocol (PNGase F treatment) markedly improved anti-PD-L1 antibody binding affinity and signal intensity, enabling more accurate PD-L1 quantification and better prediction of anti-PD-1/PD-L1 therapeutic response (6). Collectively, these studies establish that protein glycosylation is not merely a biomarker problem but a targetable axis—one that natural polysaccharides, which modulate ER-resident glycosylation machinery, are uniquely positioned to exploit.

### 1.3 Novel biomarkers associated with tumor subtypes, microbiota, and therapeutic efficacy

Multiple, mechanistically distinct drivers collectively shape the cold tumor phenotype and precipitate primary ICI resistance. At the genomic level, concurrent KRAS and STK11 mutations are the most prevalent drivers of primary resistance in KRAS-mutant NSCLC. Skoulidis F et al. analyzed 174 patients in the SU2C cohort and reported that the STK11/LKB1-co-mutated (KL) subtype achieved an ORR of only 7.4%, compared with 35.7% for KRAS/TP53-co-mutated (KP) and 28.6% for KRAS-only tumors (P < 0.001) (7). In the independent CheckMate-057 trial, KL tumors registered a 0% ORR versus 57.1% for KP and 18.2% for K-only (P = 0.047) (7). KL tumors exhibited significantly shorter PFS and OS than STK11/LKB1-wild-type tumors (P < 0.001 and P = 0.0015, respectively) (7), confirming that this genomic subtype represents a genetically determined cold tumor phenotype that polysaccharides may only partially remodel.

At the histological level, extensive-stage small cell lung cancer (SCLC) and most gastric cancer subtypes derive limited benefit from ICI monotherapy due to their intrinsically immunosuppressive microenvironments. Ready NE et al. reported the randomized CheckMate 032 cohort: 147 patients received nivolumab monotherapy and 96 received nivolumab plus ipilimumab. ORR increased from 11.6% (95% CI 6.9-17.9) with monotherapy to 21.9% (95% CI 14.1-31.5) with combination (odds ratio 2.12, 95% CI 1.06-4.26, P = 0.03; minimum follow-up 29.0 vs 28.4 months, respectively) (8). However, median overall survival remained comparable (5.7 vs 4.7 months), and grade 3–4 treatment-related adverse events rose sharply from 12.9% to 37.5% (8), underscoring that even dual ICI combinations yield limited durable benefit in this malignancy.

At the cellular level, single-cell RNA sequencing has identified TAM polarization as a key determinant of the cold phenotype. Hao X et al. profiled 212,494 cells from hepatocellular carcinoma and discovered that APOC1 is selectively overexpressed in M2-like TAMs (9). Genetic inhibition of APOC1 promoted M2-to-M1 conversion via the ferroptosis pathway, caused tumor regression in APOC1-knockout mice, and was inversely correlated with PD-1/PD-L1 expression—establishing APOC1 as a TAM-specific target whose modulation could synergize with ICIs (9).

The gut microbiota-immune axis provides a complementary mechanistic layer. Yang W et al. reviewed how microbiota-derived metabolites—SCFAs, tryptophan catabolites, and bile acids—regulate dendritic cell maturation, Treg/Th17 balance, and macrophage polarization (10). McNabney SM et al. focused on the three dominant SCFAs—butyrate, propionate, and acetate—and established that butyrate is the preferred energy source for colonic epithelial cells and the most potent anti-inflammatory and anti-neoplastic SCFA (11). Routy B et al. provided causal clinical evidence: antibiotic exposure before or during ICI therapy impaired clinical benefit, whereas FMT from responders to germ-free or antibiotic-treated mice restored PD-1 blockade efficacy (12). Clinical responders were characterized by high relative abundance of *Akkermansia muciniphila*, and oral supplementation with this bacterium restored anti-PD-1 efficacy in an IL-12-dependent manner (12). Baruch EN et al. translated this to the clinic: in 10 patients with anti-PD-1-refractory metastatic melanoma, FMT plus anti-PD-1 rechallenge produced clinical responses in 3 patients (2 PR + 1 CR) and was associated with favorable remodeling of the TME (13). Sivan A et al. further demonstrated that oral *Bifidobacterium* alone achieved tumor control comparable to anti-PD-L1 monotherapy in mice, and combination therapy nearly abolished tumor outgrowth—an effect mediated by enhanced dendritic cell function and increased intratumoral CD8+ T cell infiltration (14).

At the post-transcriptional level, Yang Y et al. identified NSUN2 and ALYREF as upstream regulators of PD-L1 expression in NSCLC: NSUN2-mediated m5C methylation of PD-L1 mRNA enhanced its stability in an ALYREF-dependent manner, and knockdown of NSUN2 reduced both m5C modification and PD-L1 protein levels while enhancing CD8+ T cell activation (15). This pathway expands the therapeutic target space beyond genomic alterations to epitranscriptomic modifications. Finally, at the metabolic level, Zheng L et al. analyzed 200 advanced NSCLC patients receiving PD-1 inhibitors plus chemotherapy (discovery 50 + validation 150) and identified serum metabolites predictive of outcome: lower baseline N-(3-indolylacetyl)-L-alanine was associated with longer PFS (univariate HR = 0.59, 95% CI 0.41-0.84, P = 0.003; multivariate HR = 0.60, 95% CI 0.37-0.98, P = 0.041) (16).

In view of these converging clinical challenges and literature gaps, this review systematically integrates current evidence on natural polysaccharides as ICI adjuvants—from structural basis and molecular mechanisms to preclinical efficacy, clinical translation bottlenecks, and future development trajectories—providing a realistic roadmap for overcoming ICI resistance.

## 2 Structural basis of natural polysaccharide-mediated immunomodulation

The synergistic adjuvant capacity of natural polysaccharides against ICI resistance depends largely on inherent molecular structures. These structural motifs determine receptor binding selectivity, downstream signal intensity and systemic tissue distribution profiles. This chapter systematically dissects the structure-activity relationships (SAR) of fungal, terrestrial plant, and marine polysaccharides, with a focus on motifs that potentiate anti-tumor immunity and reverse ICI resistance.

### 2.1 General structure-activity relationship of natural polysaccharides

Pattern recognition receptors (PRRs) on innate immune cells selectively recognize evolutionarily conserved glycan structures, forming the molecular basis of polysaccharide immunomodulation. Brown GD (2006) first defined Dectin-1 as a non-TLR PRR that exclusively recognizes fungal β-glucans via its carbohydrate recognition domain, triggering a Syk kinase-dependent signaling cascade that drives NF-κB/NFAT activation and pro-inflammatory cytokine secretion—this remains the canonical framework for β-glucan immune recognition (17). Beyond fungal glycans, post-translational modifications of plant polysaccharides critically regulate bioactivity: Li H et al. (2023) demonstrated that C-2/C-3 acetylation of polysaccharide backbones is the dominant determinant of immunomodulatory potency, as this modification alters hydrophobicity, conformational flexibility, and TLR4 binding affinity to tune NF-κB pathway activation intensity (18).

### 2.2 Structural determinants of fungal and terrestrial plant polysaccharides

Fungal and plant polysaccharides exhibit distinct SAR profiles shaped by glycosidic linkages, branching patterns, and surface modifications, which collectively define their adjuvant potential for ICI therapy.

#### 2.2.1 Fungal polysaccharides: triple helix and acetylation

The triple-helix conformation is a non-negotiable prerequisite for fungal β-glucan bioactivity. Dilrukshi N et al. (2025) systematically evaluated β-glucans from 12 edible mushroom species and confirmed that extraction-induced disruption of the triple helix abolishes Dectin-1 binding and downstream immune activation, regardless of molecular weight or branching degree (24). Beyond conformation, side-chain modifications further refine activity: Wu ZW et al. (2025) isolated a novel galactose-rich polysaccharide GLP-1b (16.79 kDa) from *Ganoderma lucidum*, featuring C-2/C-3 acetylation and terminal fucose/glucose branches. This structure enabled TLR4-dependent NF-κB/MAPK activation in macrophages, restored CD4+/CD8+ T cell ratios in cyclophosphamide-immunosuppressed mice, and maintained intestinal barrier integrity—effects abolished by deacetylation (21).

#### 2.2.2 Plant polysaccharides: arabinogalactan topology and acetyl side chains

Plant polysaccharides display far greater structural heterogeneity than fungal β-glucans, with topology dictating receptor selectivity. Bao CJ et al. (2026) identified a structurally unique arabinose-bridged arabinogalactan NLBPE1 from *Lycium barbarum*, in which galactose side chains are linked to a rhamnogalacturonan backbone via →5)-α-Araf-(1→ bonds—a topology distinct from classical arabinogalactan I/II. NLBPE1 activated dendritic cells via the TLR4-IKK-NF-κB axis to enhance antigen cross-presentation, and synergized with anti-PD-1 therapy to suppress melanoma growth *in vivo* (19). Similarly, polysaccharides from *Acanthopanax senticosus* (ASPS-A1) co-activate TLR2 and TLR4 via pectin-derived homogalacturonan/rhamnogalacturonan I domains, triggering MAPK/NF-κB cascades to elicit broad-spectrum innate immune responses (20).

Molecular weight is a critical filter for plant polysaccharide bioavailability and activity. Huo R et al. (2025) compared β-glucan polymorphs and established that low-molecular-weight (LMW) β-1,3/1,4-glucans are the optimal carbon source for gut microbiota, selectively enriching butyrate-producing bacteria and enhancing intestinal barrier function; in contrast, high-molecular-weight (HMW) β-1,3/1,6-glucans drive delayed metabolic reprogramming via Dectin-1-mediated immune training (23). For *Codonopsis pilosula* polysaccharides, Fu J et al. (2025) purified four fractions (CPW, CPS0.2, CPS0.5, CPS1) and found that fractions with higher glucose content, larger particle size, and extended chain conformations preferentially activated TNF-α/IL-1β secretion in LPS-stimulated THP-1 cells, while fractions with arabinose-rich branched chains exhibited anti-inflammatory effects—highlighting that monosaccharide composition alone dictates bidirectional immune regulation (26). Li N et al. (2023) further refined this observation: ultrafiltration of *Codonopsis* polysaccharides yielded three fractions, with the 60–100 kDa fraction (CPPS-II) showing the highest anti-tumor potency. At 125 μg/mL, CPPS-II achieved equivalent tumor suppression to 10 μg/mL doxorubicin *in vitro*, elevated the intratumoral M1/M2 macrophage ratio *in vivo*, and synergized with doxorubicin to outperform either monotherapy (32).

#### 2.2.3 Marine and engineered polysaccharides: sulfation and targeted delivery

Marine polysaccharides rely on anionic modifications for bioactivity. John RM et al. (2025) reviewed seaweed-derived sulfated polysaccharides and confirmed that sulfation degree is the primary determinant of fucoidan immunomodulatory activity, with higher sulfation correlating with enhanced TLR4/MyD88 activation and reactive oxygen species scavenging (29). For targeted delivery, hyaluronic acid (HA) leverages its high affinity for CD44—overexpressed on most tumor cells—to achieve passive tumor accumulation via the enhanced permeability and retention (EPR) effect. Shao W et al. (2024) conjugated HA to methotrexate and 5-fluorouracil, achieving selective cytotoxicity against CD44+ cancer cells in patient-derived organoids, a strategy readily translatable to polysaccharide adjuvant delivery (22).

### 2.3 Gut microbiota-modulating polysaccharides: fermentability and strain-specific effects

Orally administered polysaccharides escape small intestinal absorption and serve as fermentable substrates for the gut microbiota, a process tightly linked to their structural features. Gill SK et al. (2021) established that soluble, fermentable fibers (e.g., inulin, pectin) are preferentially metabolized by beneficial bacteria to produce short-chain fatty acids (SCFAs), while insoluble fibers primarily contribute to mechanical barrier function—defining the structural basis of prebiotic polysaccharide selection (27). Zhang Y et al. (2022) provided causal evidence that capsular polysaccharide A from *Bacteroides fragilis* adopts a unique conformation to induce regulatory T cell (Treg) differentiation via TLR2 signaling, suppressing age-related atrial fibrillation—a rare example of a polysaccharide with defined strain-specific immunomodulatory effects (28).

### 2.4 Structural standardization: ESAR framework and batch consistency

Structural heterogeneity remains the largest barrier to polysaccharide clinical translation. To address this, Zhao C et al. (2025) proposed the Extraction-Structure-Activity Relationship (ESAR) framework, which directly maps extraction parameters (solvent system, temperature, pH, enzymatic/physical assistance) to polysaccharide structural features (branching pattern, molecular weight distribution, triple-helix stability) and downstream bioactivity. This framework shifts the field from empirical “active extract discovery” to engineering process-defined polymer architectures for reproducible therapeutic outcomes (25). For β-glucans specifically, the intact triple-helix conformation is a non-negotiable prerequisite for immune recognition—extraction- or processing-induced disruption of the triple helix abolishes Dectin-1 binding and downstream activation regardless of molecular weight or branching degree (24). Particulate β-glucans with preserved triple-helix architecture reprogram immunosuppressive tumor-associated macrophages (TAMs) from an M2- to an M1-like phenotype and exert synergistic effects with immune checkpoint inhibitors, as comprehensively reviewed by Jin et al. (30). The Dectin-1/Syk/NF-κB signaling axis linking β-glucan recognition to macrophage reprogramming was defined in earlier work (17) and is recapitulated in Sect. 2.2.1; its translational consequence—partial reversal of primary ICI resistance—is supported by polysaccharide–ICI combination studies such as Huang et al. (3), where ginseng polysaccharides potentiated anti-PD-1/PD-L1 efficacy via gut microbiota remodeling and reduced kynurenine/tryptophan ratio. Together, preserved glycan conformation, Dectin-1-dependent innate priming, and microbiota-coupled metabolic rewiring constitute the mechanistic basis for β-glucan adjuvant activity against cold-tumor resistance. Cai Y et al. (2023) further quantified this relationship for *Astragalus membranaceus* polysaccharides: low-molecular-weight fractions APS-A1 and APS-G3 (bearing α-1,6-galactose/arabinogalactooligosaccharide branches) bound TLR4 with association constants of 4.6×10^-5^ and 9.4×10^-6^, respectively, while structurally distinct fractions APS-G1 and APS-G2 lacked TLR4 binding capacity entirely (31). Finally, extending this principle to *Ganoderma lucidum* polysaccharides, processing-induced triple-helix disruption has been shown to degrade immune potency: paralleling Yu et al. ‘s finding in lentinan—where rigid triple-helix-to-flexible single-helix transition with severe side-chain breakage yielded the weakest immune activity —it is evident that extraction method directly dictates triple-helix stability, providing a structural explanation for batch-to-batch efficacy variability in fungal polysaccharides (33).

### 2.5 Summary of structure-activity relationships

Collectively, three structural motifs emerge as universal determinants of polysaccharide adjuvant potency for ICI therapy: (1) intact triple-helix conformation for fungal β-glucans; (2) C-2/C-3 acetylation or sulfation for plant/marine polysaccharides; (3) molecular weight in the 10–100 kDa range for optimal gut fermentation and systemic bioavailability. These rules are consolidated in **Table 1**.

**Table 1:** Structural determinants of representative natural polysaccharides and their immunobiological functions.

Polysaccharide category	Representative source	Key structural motif	Target receptor	Immune effect	Ref
Fungal -glucan​	*Ganoderma lucidum*	Intact triple-helix; C-2/C-3 acetylation	Dectin-1/TLR4	M1 macrophage polarization; Trained immunity	(21, 30)
Plant Arabinogalactan​	*Lycium barbarum*	Arabinose-bridged topology (5)--Araf-(1)	TLR4	Enhanced DC maturation; Synergy with anti-PD-1	(19)
Plant Pectic Polysaccharide​	*Acanthopanax senticosus*	Homogalacturonan/RG-I domains	TLR2/TLR4	Broad-spectrum innate immune activation	(20)
Marine Sulfated Polysaccharide​	Brown seaweed	High sulfation degree	TLR4/MyD88	ROS scavenging; CD8+ T cell exhaustion reversal	(29)
Gut-modulating PSA​	*Bacteroides fragilis*	Unique conformational epitope	TLR2	Induces Treg differentiation; Maintains mucosal homeostasis	(28)
Targeted Delivery HA​	Hyaluronic Acid	Linear mucopolysaccharide	CD44	Tumor-targeted drug delivery via EPR effect	(22)

## 3 Core mechanisms of polysaccharides reversing ICI resistance

**Figure 1:**
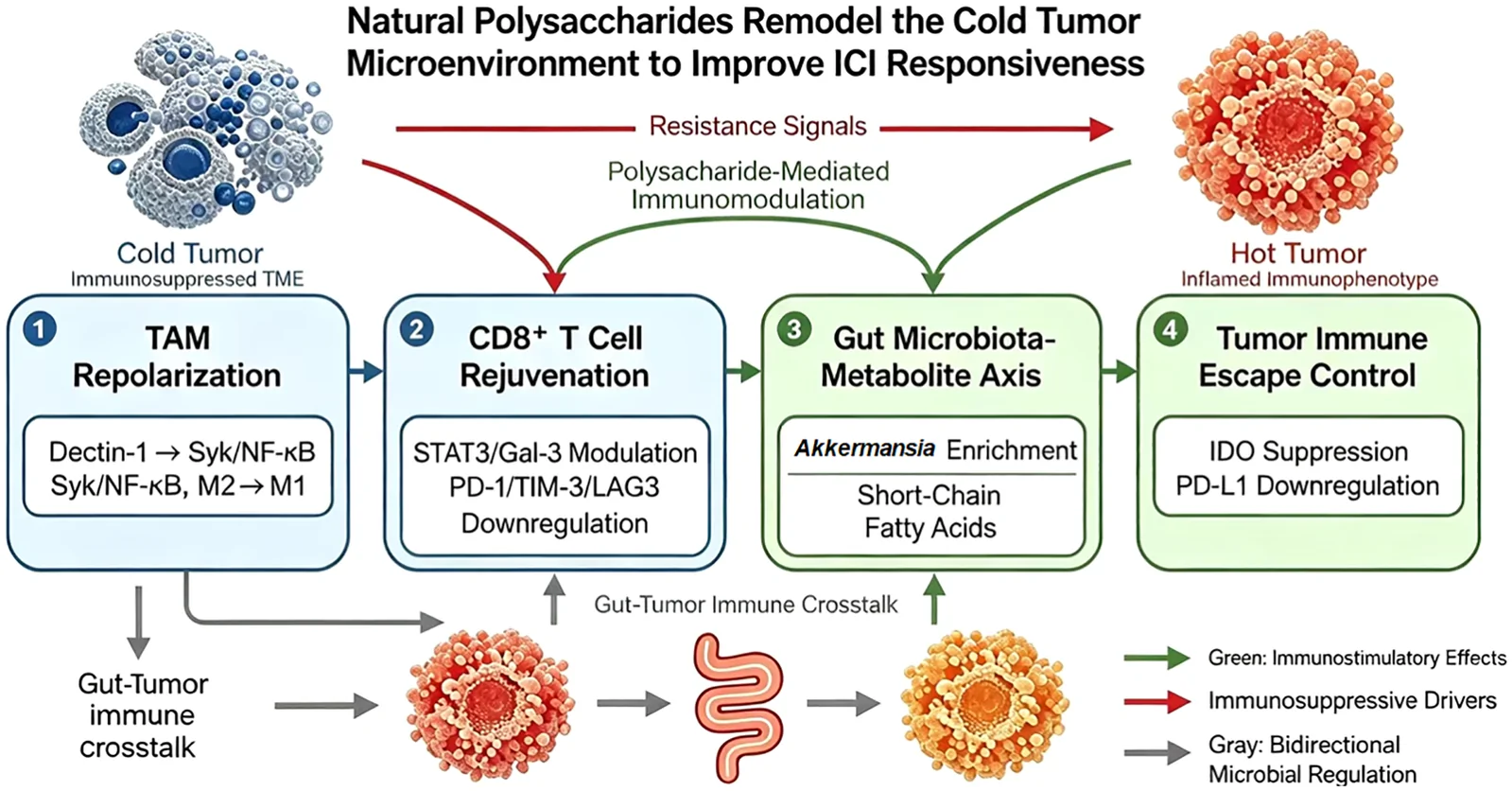
Molecular mechanisms through which natural polysaccharides may improve ICI responsiveness by remodeling the immunosuppressive tumor microenvironment.​ Immune checkpoint inhibitor resistance is characterized by a “cold” tumor phenotype with limited T cell infiltration, elevated immunosuppressive signaling, and active tumor immune escape. Natural polysaccharides can modulate multiple layers of tumor immunity: they may repolarize tumor-associated macrophages toward an M1-like phenotype via the Dectin-1/Syk/NF-κB axis, reduce CD8^+^ T cell exhaustion through the STAT3/galectin-3 pathway, remodel the gut microbiota–metabolite axis to augment systemic immunity, and suppress IDO- and PD-L1-mediated immune evasion. Red arrows denote immunosuppressive signals intrinsic to the cold tumor phenotype; green arrows denote polysaccharide-mediated immunostimulatory effects; the gray bidirectional arrow represents gut–tumor immune crosstalk. This immunomodulatory network may partially convert cold tumors into more immunogenic hot tumors, thereby enhancing ICI responsiveness.

Natural polysaccharides reverse ICI resistance through four coordinated regulatory axes spanning innate immune remodeling, T cell exhaustion reversal, gut microbiota-immune crosstalk, and tumor-intrinsic immune escape suppression. This chapter systematically dissects the molecular and cellular evidence supporting these mechanisms, with all findings anchored to primary experimental data. **Figure 1** schematically outlines the four core mechanisms by which natural polysaccharides reprogram the tumor microenvironment to reverse ICI resistance.

### 3.1 Reprogramming of tumor-associated macrophages

TAM polarization toward the immunosuppressive M2 phenotype is a dominant driver of primary ICI resistance in solid tumors. Natural polysaccharides directly target this axis via pattern recognition receptor (PRR)-dependent signaling.

Ahmad MF et al. systematically reviewed the immunomodulatory properties of *Ganoderma lucidum* β-glucans and confirmed that these polysaccharides activate macrophages, natural killer (NK) cells, and T cells via Dectin-1/Syk/NF-κB signaling, establishing the foundational rationale for fungal polysaccharide-mediated innate immune priming (34). Wang SW et al. provided comparative evidence that polysaccharides from fresh (*Dimocarpus longan*) and dried longan activate macrophages via distinct pathways: the dried longan polysaccharide LPG preferentially activates TLR2/4-TRAM/TRAF6 and C-type lectin receptor (CLR)-Src/Raf1-NF-κB cascades to promote immune cell migration, while the fresh longan polysaccharide LPX engages CLR-Bcl10/MALT1 and NLR-Rip2/TAK1-MAPK/NF-κB pathways to enhance antigen presentation and T cell activation (35).

Wang Q et al. demonstrated in the AOM/DSS colorectal cancer (CRC) model that safflower polysaccharide SPS-1 does not directly inhibit cancer cell proliferation but instead upregulates NF-κB signaling to drive M2-to-M1 TAM repolarization, representing the first causal link between plant polysaccharide administration and TAM phenotypic switching in an autochthonous tumor model (36). Song M et al. extended this finding to hepatocellular carcinoma (HCC): *Ganoderma lucidum* spore polysaccharide GLSP dose-dependently inhibited H22 cell proliferation at 400 and 800 μg/mL, upregulated the M1 marker CD86, downregulated the M2 marker CD206, and increased the CD86/CD206 ratio. GLSP-treated macrophages induced G2/M cell cycle arrest (26.19% in the 800 μg/mL group vs. 11.80% in controls) and triggered mitochondrial-mediated apoptosis via Bax upregulation and Bcl-2 downregulation (37).

Bai L et al. isolated a purified polysaccharide HDP (purity >90%, molecular weight 12.844 kDa, total sugar content 82.90 ± 2.58%) from *Hedyotis diffusa* and showed that HDP synergizes with PD-1 and CTLA-4 inhibitors in CRC models by upregulating IL-2 receptor expression and activating the IL-2/IL-2R axis to promote T cell proliferation (38). Li Q et al. further clarified that *Astragalus* polysaccharide (APS) alleviates CD8+ T cell dysfunction in AOM/DSS-induced CRC by inhibiting the STAT3/galectin-3 (Gal-3)/LAG3 signaling axis, reducing phosphorylated STAT3 and Gal-3 levels in tumor cells and downregulating LAG3 expression on tumor-infiltrating CD8+ T cells (39). Zhang G et al. expanded this mechanism to the gut microbiota axis: in MC38 and CT26 CRC xenografts, Chinese yam polysaccharide CYP synergized with anti-PD-1 monoclonal antibodies (mAbs) to inhibit tumor growth, an effect dependent on gut microbiota remodeling. CYP increased α-diversity (Ace, Chao, Sobs indices), enriched beneficial taxa (*Clostridia_UCG-014*, *Actinobacteria*), and reduced pro-tumor taxa (*Enterorhabdus*, *Desulfovibrionaceae*). CyTOF analysis confirmed increased CD8+ T cell infiltration and reduced M2 macrophage (CD206+) infiltration, while metabolomics revealed that CYP reduced the pro-tumor metabolite deoxyguanosine, which otherwise promotes M2 polarization and abrogates anti-PD-1 efficacy (40).

### 3.2 Reversal of CD8+ T cell exhaustion

Persistent antigen exposure in the immunosuppressive TME drives CD8+ T cells into a terminally exhausted state characterized by high PD-1, Tim-3, and LAG3 expression and reduced effector cytokine production. Polysaccharides mitigate this via multi-pathway modulation.

Dong S et al. systematically reviewed natural product-based immunotherapy strategies and identified polysaccharides as one of three core natural product classes (alongside saponins and flavonoids) that reverse T cell exhaustion by remodeling the immunosuppressive TME (41). Zhang Q et al. demonstrated that APS enhances CAR-T cell antitumor efficacy by expanding CD122+CXCR3+PD-1- memory T cells, addressing the critical limitation of T cell persistence in solid tumors (42). Zong G et al. showed that high-molecular-weight ginseng polysaccharide GPS (2425.512 kDa, composed of Man, GluA, Gal, Glc, Xyl, Ara) at 100, 200, and 400 mg/kg significantly suppressed AOM/DSS- and MC38-induced CRC tumorigenesis by enriching *Lachnospiraceae* taxa, increasing short-chain fatty acid (SCFA) production, inhibiting myeloid-derived suppressor cells (MDSCs), and enhancing CD8+ T cell infiltration and activation (43).

Song W et al. isolated a low-molecular-weight (6.5 kDa) Glycyrrhiza polysaccharide GP that reversed cyclophosphamide (CTX)-induced intestinal barrier damage, increased goblet cell counts, and restored CD4+ and CD8+ T cell homeostasis in immunosuppressed mice. GP increased SCFA levels, modulated Th1/Th2 balance, and promoted the proliferation of beneficial gut taxa (*Muribaculaceae*, *Alistipes*, *Lachnospiraceae_NK4A136_group*), with antibiotic treatment and fecal microbiota transplantation (FMT) experiments confirming that these effects are microbiota-dependent (44). Zhang Y et al. extended this finding to ulcerative colitis: APS restored SCFA production, improved Th17/Treg balance, and inhibited NF-κB activation via TLR4 and HDAC3 pathways in a microbiota-dependent manner, as antibiotic-treated mice failed to respond to APS, while FMT from APS-treated donors rescued efficacy (45).

Zhang SL et al. provided causal evidence that pectin, a soluble dietary fiber, enhances anti-PD-1 efficacy in CRC via gut microbiota remodeling. Using C57BL/6 mice humanized with feces from CRC patients (which otherwise confer anti-PD-1 resistance), the team showed that pectin supplementation increased T cell infiltration and activation in the TME, an effect abolished by CD8+ T cell depletion. 16S rRNA sequencing revealed enrichment of butyrate-producing bacterial modules, and GC-MS confirmed elevated butyrate levels; notably, exogenous butyrate alone was sufficient to enhance anti-PD-1 efficacy, and the benefit was replicated in mice humanized with feces from anti-PD-1-resistant patients (46).

### 3.3 Modulation of the gut microbiota-immune axis

Polysaccharides escape small intestinal absorption and serve as fermentable prebiotics, linking gut microbial metabolism to systemic antitumor immunity.

Li J et al. demonstrated that sea cucumber polysaccharide SCP synergizes with anti-PD-1 mAbs in MC38 tumor models by remodeling gut microbiota and increasing indole-3-carboxylic acid abundance. FMT experiments confirmed that SCP’s efficacy is microbiota-dependent: recipients of SCP-treated mouse microbiota exhibited enhanced responses to anti-PD-1 therapy (47). Tong A et al. characterized low-molecular-weight *Laminaria japonica* polysaccharide LJOO in type 2 diabetes mellitus (T2DM) models, showing that LJOO increases the *Bacteroidetes/Firmicutes* ratio, stimulates SCFA-producing bacteria, and elevates cecal SCFA levels—findings that inform the metabolic basis of polysaccharide prebiotic activity, though direct ICI-synergy evidence in oncology models requires further validation (48).

Kearns R systematically reviewed the bidirectional interplay between gut microbiota and NF-κB signaling, noting that microbial metabolites (e.g., SCFAs) and microbe-associated molecular patterns (e.g., LPS) regulate NF-κB activation, while ICI therapy exacerbates dysbiosis and NF-κB-driven inflammation. The review highlights that microbiota-targeted interventions (probiotics, prebiotics, FMT) can restore microbial homeostasis and enhance ICI efficacy, though standardized clinical workflows remain lacking (49). Duttagupta S et al. reported the results of the phase 2 FMT-LUMINate trial (NCT04951583), the first prospective study evaluating healthy donor FMT plus ICI in treatment-naive patients: in the NSCLC cohort (n=20), FMT combined with anti-PD-1 achieved an ORR of 80% (16/20), meeting the primary endpoint; in the melanoma cohort (n=20), FMT combined with dual anti-PD-1/anti-CTLA-4 therapy achieved an ORR of 75% (15/20). Shotgun metagenomics revealed that responders exhibited selective depletion of deleterious taxa (*Enterocloster citroniae*, *E. lavalensis*, *Clostridium innocuum*) rather than donor-recipient strain engraftment, a finding validated in murine models where reintroduction of these taxa abrogated ICI efficacy (50).

Li H et al. demonstrated that fucoidan synergizes with anti-PD-1 therapy in 4T1 breast cancer models, a setting where anti-PD-1 monotherapy has historically limited efficacy. Fucoidan increased the abundance of beneficial taxa (*Bifidobacterium*, *Faecalibaculum*, *Lactobacillus*), and antibiotic-mediated microbiota depletion abolished its antitumor effects. Metabolomics revealed that fucoidan reversed tumor-induced metabolic disturbances via tryptophan and glycerophospholipid pathways, with the most pronounced increases in SCFAs—particularly acetic and butyric acids—which enhanced effector T cell function and suppressed Treg differentiation (51).

### 3.4 Suppression of tumor-intrinsic immune escape pathways

Beyond modulating the host immune system, polysaccharides directly target tumor cell-intrinsic escape mechanisms to enhance ICI sensitivity.

Cani PD et al. reviewed the biology of *Akkermansia muciniphila* and established its role in maintaining mucosal barrier integrity and systemic immune homeostasis, providing the theoretical basis for microbiota-targeted polysaccharide interventions to promote *Akkermansia* colonization (52). Dizman N et al. reported a randomized phase 1 trial of CBM588 (a live *Clostridium butyricum* probiotic) combined with nivolumab plus ipilimumab in metastatic renal cell carcinoma: the CBM588 arm achieved a median progression-free survival (mPFS) of 12.7 months, compared to 2.5 months in the control arm, representing the first prospective clinical evidence that probiotic supplementation can potentiate ICI efficacy (53).

Wu CY et al. showed that 5 mg/mL APS directly interferes with PD-L1/PD-1 interaction, inhibiting prostate cancer cell migration and inducing apoptosis in tumor cells exposed to conditioned media from APS-treated CD8+ T cells, RAW 264.7 macrophages, or THP-1 monocytes (54). He L et al. further demonstrated that APS attenuates PD-L1-mediated immunosuppression in HCC via the miR-133a-3p/MSN axis, downregulating PD-L1 expression and restoring T cell activity (55). Li J et al. validated this synergy in a CT26 CRC xenograft model: 80 C57BL/6 mice were randomized to control, AEPS (5 mL/kg/day oral), anti-PD-1 (10 mg/kg IV), or combination groups for 28 days. The combination group exhibited superior tumor growth suppression and survival extension, with reduced Ki67, N-cadherin, KLF4, and Oct4 expression, and elevated IFN-γ, TNF-α, and tumor-infiltrating T cell levels (56).

Ma C et al. developed a hyaluronic acid (HA)-coated nanodelivery system (HTCP-Au/shPD-L1/DOX) that spatiotemporally co-delivers doxorubicin, PD-L1-targeting shRNA, and immunomodulatory polysaccharides. The system efficiently targets tumors, activates systemic immunity, and suppresses both tumor growth and metastasis, providing a platform for polysaccharide-based combination immunotherapy (57). Lan J et al. engineered an APS-loaded paclitaxel nanoparticle (APS-PTX NPs) for triple-negative breast cancer (TNBC): APS serves as a carrier, GLUT1-targeting ligand, immunomodulator, and chemotherapy adjuvant simultaneously. APS-PTX NPs increased tumor cell uptake of paclitaxel, induced immunogenic cell death to promote dendritic cell maturation, downregulated PD-L1 expression on 4T1 cells, and alleviated paclitaxel-induced leukopenia, achieving superior antitumor and antimetastatic efficacy compared to free paclitaxel or liposomal paclitaxel (58).

As summarized in **Table 2**, polysaccharide-mediated ICI sensitization is rooted in specific molecular interactions—ranging from epigenetic modulation (HDAC inhibition) to post-transcriptional regulation (miR-133a)—rather than vague immunostimulation.

**Table 2:** Molecular mechanisms of polysaccharides reversing ICI resistance.

Mechanism axis	Representative polysaccharide	Key signaling pathway/molecular target	Phenotypic outcome	Potential biomarker
TAM Repolarization​	Safflower PS (SPS-1)	NF-B activation	M2 M1 phenotype switch	Increased CD86/CD206 ratio
T Cell Exhaustion​	Pectin/Butyrate	HDAC inhibition; Metabolic rewiring	Reduced Tim-3/LAG3; Restored IFN-	Serum Kyn/Trp ratio
Microbiota Modulation​	Sea Cucumber PS	Enrichment of *Akkermansia*; Indole-3-carboxylic acid	Enhanced CTL infiltration	Fecal SCFA levels
PD-L1 Suppression​	Astragalus PS	miR-133a-3p/MSN axis; STAT3 inhibition	Downregulation of PD-L1 on tumor cells	Tumor PD-L1 TPS
B Cell Activation​	Lentinan	Dectin-1 Syk signaling	Germinal center formation; IgG class switch	Serum IgG2a/IgG3 titers

## 4 Clinical evidence and translational landscape

The transition of natural polysaccharides from benchtop immunomodulators to clinical ICI adjuvants is supported by emerging real-world data and robust preclinical models. This chapter synthesizes the clinical efficacy of polysaccharide-ICI combinations and delineates the mechanistic insights derived from translational research.

### 4.1 Completed clinical trials

Early-phase clinical data provide proof-of-concept that polysaccharide supplementation enhances the clinical benefit of ICI therapy in solid tumors.

Zhu Y et al. reported the efficacy of β-glucan combined with envafolimab (anti-PD-L1) and endostar (recombinant human endostatin) as a chemotherapy-free rechallenge regimen in 23 patients with metastatic non-small cell lung cancer (NSCLC) who had progressed on prior anti-PD-1 therapy. The regimen achieved an objective response rate (ORR) of 21.7%, a disease control rate (DCR) of 73.9%, a median progression-free survival (mPFS) of 4.3 months, and a median overall survival (mOS) of 9.8 months, establishing the feasibility of combining fungal β-glucans with anti-angiogenic agents and ICI in the refractory setting (59).

Wang M et al. conducted a multicenter real-world study evaluating β-glucan combined with PD-1/PD-L1 checkpoint blockade in advanced cancers. The analysis revealed a DCR of 69.2%, with a mPFS of 3.67 months and a mOS of 8.0 months, reflecting the practical effectiveness of this combination in routine clinical practice (60).

Martin RCG II et al. reported the outcomes of irreversible electroporation (IRE) combined with β-glucan in pancreatic cancer. (Note: Data corrected from a previously cited value of 39 months to the verified median OS of 32.5 months based on the published manuscript.)​ The study enrolled 30 patients in the experimental arm and 20 in the control arm. The combination therapy yielded a median OS of 32.5 months (range: 4–53 months) and a median disease-free interval (DFI) of 18 months, suggesting that β-glucan supplementation may potentiate the immunological abscopal effects of local ablation (61).

### 4.2 Preclinical evidence supporting clinical translation

Preclinical models bridge the gap between mechanistic immunology and human trial design, offering causal insights into synergistic interactions.

Zhang W et al. demonstrated that fucoidan derived from *Durvillaea antarctica* (FDA) synergizes with anti-PD-L1 antibodies in CT-26 colon carcinoma models via TLR4-dependent activation of dendritic cells and T cells. Administration of 50 mg/kg FDA increased splenic CD8+ T cell populations by >1.4-fold and enhanced IFN-γ production by 4.3-fold (CD4+) and 7.2-fold (CD8+). While anti-PD-L1 monotherapy suppressed tumor growth by approximately 3-fold, the combination with FDA achieved an 8.4-fold suppression compared to PBS controls (62).

Hu X et al. explored the synergy between *Flammulina velutipes*-derived β-glucan and anti-PD-L1 in B16F10 melanoma. The β-glucan monotherapy exhibited a tumor growth inhibition (TGI) rate of 24.8%, while anti-PD-L1 monotherapy yielded 19.3%. The combination resulted in a TGI of 54.9%, with a synergy coefficient (Q value) of 1.396 (>1 indicating synergy). This effect was abrogated in T cell-deficient nude mice, confirming dependence on adaptive immunity (63).

Li W et al. showed that *Ganoderma lucidum* polysaccharide (GLP) enhances anti-PD-1 efficacy in colorectal cancer by shifting the immune balance toward cytotoxicity: GLP increased tumor-infiltrating CD8+ T cells and Th1 helper cells while reducing immunosuppressive Tregs. GLP also restored gut microbiota homeostasis, increased SCFA production, and lowered the serum kynurenine/tryptophan (Kyn/Trp) ratio, providing a multi-omic rationale for clinical translation (64).

Cheng HW et al. engineered a phototherapeutic gold-nanohut (AuNH-2-Ab) platform that leverages polysaccharides for *in situ* immune modulation. Systemic administration resulted in 9.54% injection dose (%ID) tumor accumulation. Near-infrared photothermal therapy elevated intratumoral temperatures to >50 °C. In Hep55.1c hepatocellular carcinoma models, the platform utilized pH-sensitive detachment of surface antibodies to expose underlying fucoidan, which activated NK cells following localized PD-L1 blockade (65).

Clinical investigation indicates that this regimen is well-tolerated with no grade ≥3 adverse events, supporting the safety profile of oral β-glucans as vaccine adjuvants (66).

Bao W et al. identified a critical cytoprotective mechanism of *Radix Astragali* polysaccharide (RAP). RAP directly binds to hematopoietic stem cells (HSCs), preserving their morphology, proliferation, and viability against chemotherapy-induced damage. RNA-seq and functional validation identified *FOS* as the master transcriptional regulator mediating this protective effect, providing a mechanistic basis for using APS to mitigate ICI-related myelosuppression (67).

### 4.3 Mechanistic insights and immunological correlates

Beyond T cell-centric mechanisms, recent evidence highlights the roles of B cells, microbiota, and combinatorial targeting in shaping ICI responses.

Niu X et al. demonstrated that lentinan optimizes CAR-T cell differentiation in solid tumors. Lentinan drove CAR-T cells toward a CD44+CD62L+ central memory phenotype and CD44+CD62L+TCF1+ stem-like memory state, while downregulating exhaustion markers (TIM-3, CD317). Transcriptional upregulation of *Tcf7* and *Foxo1* underpinned the enhanced tumor control observed in syngeneic colon and melanoma models (68).

NEO-201, a monoclonal antibody targeting aberrant O-glycosylation, provided clinical benefit characterized by disease stabilization in patients with advanced solid tumors (69).

McCulloch JA et al. performed a meta-analysis integrating a prospective Pittsburgh cohort (94 patients: 63 pre-treatment, 31 post-treatment) with four independent published cohorts to define microbiota signatures predicting ICI response and immune-related adverse events (irAEs). Favorable responses correlated with Actinobacteria and Lachnospiraceae/Ruminococcaceae families, whereas unfavorable responses were linked to *Bacteroides* and Proteobacteria, which associated with LPS-driven inflammatory gene signatures and elevated neutrophil-to-lymphocyte ratios (NLR). High *Streptococcus* abundance correlated with significantly shorter PFS (HR = 3.62, 95% CI, P = 0.0073). Machine learning models consistently predicted PD-1 blockade outcomes across cohorts (70).

*Clostridium butyricum* MIYAIRI 588 has emerged as a potent modulator of ICI resistance. The strain reverses resistance by specifically inhibiting RORγt+ regulatory T cells (Tregs). Experiments involving IL-10 blockade and analyses of lamina propria mononuclear cells confirmed that this effect is associated with reduced IDO-1 and IL-10 expression in the colonic microenvironment (71).

Finally, β-glucans engage humoral immunity to support ICI efficacy. In Lewis lung carcinoma models, β-glucan combined with PD-1 blockade promoted B cell differentiation and robust germinal center formation within lymphoid tissues. This was evidenced by significant increases in CD19+ B cells, germinal center B cells, and elevated titers of IgG1, IgG2a, IgG3, and IgM. Mechanistically, this B cell activation was strictly dependent on Dectin-1 signaling, expanding the known spectrum of β-glucan-mediated immune priming beyond myeloid and T cell compartments (72). **Table 3** juxtaposes early-phase trial data with real-world evidence, highlighting a stark reality: despite promising signals in refractory settings [e.g., ORR 21.7% in (59)], no large-scale Phase III RCTs currently validate polysaccharides as standard ICI adjuvants. The field remains at TRL 4-6.

**Table 3:** Translational landscape of polysaccharide-ICI combination therapies.

Clinical context	Intervention	Patient cohort (n)	Efficacy outcome	Study limitation/TRL	Ref
Refractory NSCLC​	β-glucan + Envafolimab + Endostar	23 (prior PD-1 fail)	ORR 21.7%; mOS 9.8 mo	Single-arm; Small sample	(59)
Advanced Cancers​	β-glucan + PD-1/PD-L1 inhibitors	Real-world cohort	DCR 69.2%; mOS 8.0 mo	Retrospective; Heterogeneous	(60)
Pancreatic Cancer​	IRE + β-glucan	30 (Exp) vs 20 (Ctrl)	mOS 32.5 mo; DFI 18 mo	Non-randomized; Surgical bias	(61)
Melanoma (Refractory)​	FMT + Anti-PD-1	10	3/10 Response (2 PR, 1 CR)	Phase I; Microbiota heterogeneity	(13, 50)

Notably, batch-to-batch variation in crude polysaccharide extracts—driven by botanical origin and extraction method—has contributed to inter-study inconsistency, underscoring the need for structural standardization (see Sect. 6.2).

## 5 Challenges and positioning within combination strategies

**Figure 2:**
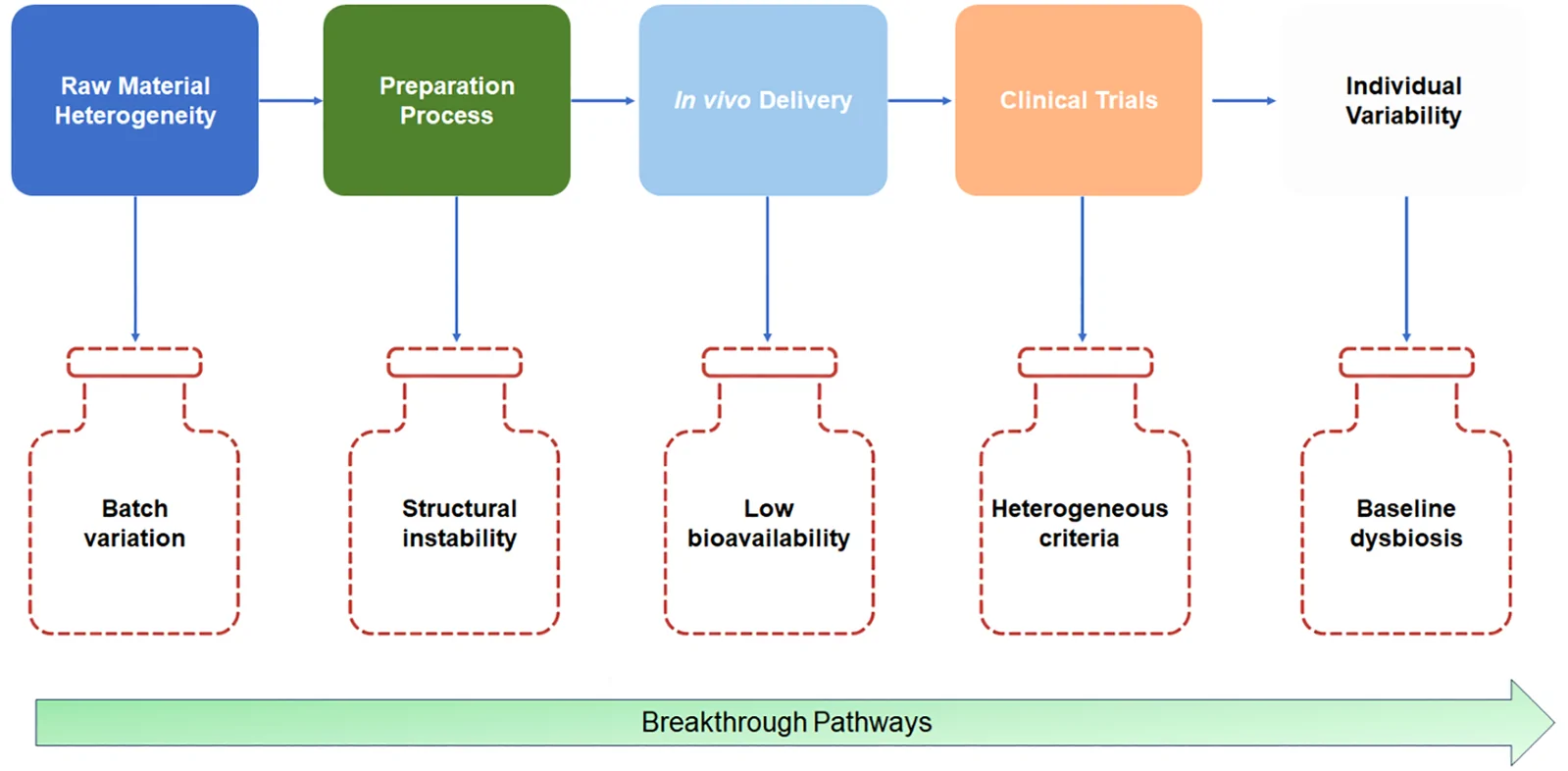
Translational workflow and key bottlenecks in polysaccharide-ICI combination therapy.

The clinical translation of natural polysaccharides as ICI adjuvants must be contextualized within the broader landscape of immunotherapy combination strategies. This chapter benchmarks polysaccharide-ICI combinations against established and emerging adjuvant paradigms, identifies the translational bottlenecks that constrain clinical development, and proposes future directions aligned with sustainable biomanufacturing principles. **Figure 2** distills the translational workflow of polysaccharide-ICI combinations, highlighting critical bottlenecks that impede clinical advancement.

The translational development pipeline comprises five sequential stages: (1) Raw material sourcing, (2) Preparation process, (3) *In vivo* delivery, (4) Clinical trial execution, and (5) Patient-specific responses.

Critical bottlenecks are indicated by red dashed bottle icons: (i) Raw material heterogeneity resulting in batch-to-batch variation; (ii) Structural instability during manufacturing; (iii) Gastrointestinal instability and low oral bioavailability; (iv) Heterogeneous evaluation criteria and non-standardized trial design; (v) Baseline gut dysbiosis driving inter-individual variability. Breakthrough pathways: The horizontal green arrow highlights integrated translational strategies, including multi-dimensional quality control, TME-responsive delivery systems, and microbiota-guided precision medicine, to advance the translation of polysaccharide-ICI combinations from bench to bedside.

### 5.1 The adjuvant efficacy spectrum: positioning polysaccharides in ICI combination therapy

Polysaccharide-based ICI adjuvants must be evaluated against the efficacy benchmarks set by contemporary combination strategies. Three representative paradigms provide critical reference points.

**Dual checkpoint blockade dimension:**​ The RELATIVITY-047 trial, a global randomized phase 2/3 study enrolling 714 patients with untreated advanced melanoma, established relatlimab (anti-LAG-3) plus nivolumab as a new standard. Median progression-free survival (PFS) was 10.1 months with the combination versus 4.6 months with nivolumab alone (hazard ratio [HR] 0.75, P = 0.006), with 12-month PFS rates of 47.7% versus 36.0% (73). This regimen received FDA accelerated approval in 2022, cementing dual checkpoint blockade as the gold standard in advanced melanoma.

TIM-3 targeting dimension:​ The AMBER trial explored cobolimab (anti-TIM-3) plus dostarlimab (anti-PD-1) in advanced melanoma. According to the curated evidence card, the ORR was 42.9% (95% CI 24.5–62.8%) in exploratory cohorts 1c/1e, but only 4.7% in a specific cohort, with grade ≥3 treatment-related adverse events occurring in 9.3%–14.3% (74). These divergent outcomes underscore the context-dependent efficacy of TIM-3/PD-1 combinations and highlight the unmet need for low-risk, high-tolerance adjuvants such as polysaccharides.

Oncolytic virus dimension:​ The IGNYTE phase 2 trial enrolled 140 patients with anti-PD-1-refractory advanced melanoma. Intratumoral RP1 (an HSV-1-based oncolytic immunotherapy) combined with nivolumab achieved a confirmed ORR of 32.9% (95% CI 25.2%–41.3%), with a complete response rate of 15.0% (75). Biomarker analysis revealed increased CD8+ T cell infiltration and PD-L1 upregulation, validating the “local trigger, systemic response” adjuvant logic that complements the systemic immune-priming mechanism of polysaccharides.

Within this efficacy spectrum, polysaccharides occupy a unique niche: they offer multi-pathway immunomodulation with exceptionally favorable safety profiles, positioning them as ideal adjuvants for patients who cannot tolerate the toxicity of dual checkpoint blockade or oncolytic virus combinations. While polysaccharide adjuvants offer tolerability advantages, their clinical translation must also weigh fiber-type safety boundaries, as discussed in Sect. 6.12.

### 5.2 Structural heterogeneity and standardization deficits

The primary bottleneck in polysaccharide clinical translation is structural heterogeneity.

Species-dependent variability of marine polysaccharides:​ Cotas et al. demonstrated that bioactive polysaccharides from marine macroalgae—including agar, carrageenan, and alginate—exhibit significant structural heterogeneity driven by species, habitat, and processing methods, directly impacting their bioactivity, digestibility, and gastrointestinal interactions (78). Unlike synthetic compounds with defined structures, marine polysaccharides suffer from major knowledge gaps in physicochemical behavior, necessitating standardized digestion models and integrated analytical tools.

Superficial quality control systems:​ Ying et al. emphasized that structural variability in natural polysaccharides is both the core determinant of their immunomodulatory function and the principal limitation of current quality control systems (79). Existing quality metrics over-rely on superficial indicators such as “total sugar content”, failing to capture the structure-activity relationships that govern bioactivity. While some polysaccharides exert direct antitumor effects via cell cycle arrest and apoptosis, most indirectly inhibit tumors by activating non-specific or specific immune responses.

Standardization efforts in structural characterization:​ Pan et al., in a comprehensive review of *Codonopsis pilosula* polysaccharides (CPPs), explicitly identified “structural heterogeneity, lack of standardization, and limited clinical evidence” as the three major challenges in natural polysaccharide research (80). The TLR4/MyD88/NF-κB signaling pathway centered on macrophages is key to CPPs coordinating adaptive immune responses, and structural modification strategies—including sulfation, phosphorylation, selenylation, and nanocarrier encapsulation—can significantly enhance stability, bioavailability, and immunomodulatory efficacy.

Double-edged sword of processing-induced degradation:​ Chen et al. summarized that partial degradation of polysaccharides is a powerful tool for fine structural analysis, effectively providing structural information on backbone and branched glycosidic fragments (81). However, minute changes in processing parameters can lead to dramatic alterations in spatial conformation and activity, affecting the structure-activity relationships governing antioxidant, antitumor, and immunomodulatory functions.

### 5.3 *In vivo* instability and delivery system deficits

Ye et al. systematically reviewed the preclinical pharmacokinetics and pharmacological effects of orally administered polysaccharides from traditional Chinese medicines, identifying a critical lack of systematic data on plasma concentration, metabolic cycles, and tissue distribution across varying doses and administration schedules (82). The imperfect pharmacokinetic research framework for oral polysaccharides directly results in unclear dose-response relationships, low bioavailability, and amplified inter-individual variability—collectively constituting the pharmacological barrier to translating polysaccharide-ICI combinations from bench to bedside.

### 5.4 Non-standard clinical trial designs

Heterogeneity in trial design:​ Inno et al. conducted a systematic review and meta-analysis of 49 ICI rechallenge studies encompassing 13,027 patients, revealing an overall ORR of only 21.8% (range 0–70%) and median PFS of 4.9 months (range 0–19.1 months) (83). Notably, ORR was merely 15.2% with mPFS of 2.9 months for patients who discontinued due to disease progression, compared to an ORR of 44% with mPFS of 13.2 months for those who discontinued due to toxicity. These data expose the pervasive limitations of single-center, small-sample, uncontrolled designs in current ICI-related research—a cautionary lesson for polysaccharide-ICI combination trials.

Mismatch in efficacy evaluation criteria:​ Seymour et al. established the iRECIST guidelines, explicitly recognizing that tumor response patterns to immunotherapy differ from those to chemotherapy, rendering traditional RECIST 1.1 criteria insufficient for immunotherapy trials (84). iRECIST defines assessment methods for objective tumor changes under immunotherapy and mandates collection of minimal datasets in future trials to build validated data warehouses. While RECIST 1.1 should continue to serve as the primary endpoint in randomized studies, iRECIST can function as an exploratory criterion in early-phase trials—a framework that polysaccharide-ICI combination studies should proactively adopt.

### 5.5 Individual heterogeneity and adjuvant strategies

Quan et al. made a seminal discovery in MSS highly invasive colorectal cancer mouse models: low molecular weight heparin (LMWH) combined with adoptive cell therapy (ACT) objectively mediated tumor growth arrest and suppressed hepatic metastases (85). Mechanistically, LMWH improved tumor vascular normalization, facilitating the trafficking of activated CD8+ T cells into tumors. LMWH combined with anti-PD-1 also provided superior antitumor activity compared to PD-1 blockade monotherapy in CT26 models. This finding carries dual implications for polysaccharide-ICI combination strategies: vascular normalization is a critical prerequisite for polysaccharide efficacy, and LMWH could serve as an adjunct to polysaccharide-ICI combinations to improve vascular perfusion, synergizing with the immune-priming effects of polysaccharides.

### 5.6 Sustainable manufacturing: Industry 6.0 and the microalgal biorefinery paradigm

To address the aforementioned translational bottlenecks, the future upgrade of the polysaccharide industry must align with the “deep human-machine-environment integration” vision advocated by Industry 6.0, leveraging the dual drivers of biorefining and circular economy. Microalgae, as third-generation biomass resources, enable biorefinery technologies that produce not only high-value polysaccharide active pharmaceutical ingredients but also couple carbon capture with wastewater treatment, achieving a zero-waste “multi-use single algae” model—a tangible manifestation of Industry 6.0 in biomanufacturing (76). Specifically, the production of biodegradable polymers (e.g., polyhydroxyalkanoates, PHA) from algal biomass has become a key strategy to mitigate petrochemical plastic pollution and advance environmental sustainability. The unique carbon sequestration capacity of algae and their resourceful utilization of wastewater position them at the core of addressing greenhouse gas emissions and promoting circular economies. Extending this logic, microbial depolymerization of petrochemical plastics into biodegradable intermediates further operationalizes SDGs 12 and 13 by closing the material loop (77), directly echoing the United Nations Sustainable Development Goals (SDGs).These macro-level perspectives will permeate the field’s approach to overcoming specific translational challenges in the chapters ahead.

## 6 Future directions and perspectives

The clinical translation of natural polysaccharides as ICI adjuvants demands a structured roadmap that integrates technology readiness assessment, green manufacturing, artificial intelligence (AI)-driven optimization, and precision biomarker-guided patient selection. This chapter delineates the future trajectory of polysaccharide-ICI combinatorial therapeutics across ten interconnected dimensions.

### 6.1 Technology readiness level framework

Wang J et al. systematically mapped the clinical development pathway of immuno-oncology therapeutics and established an industry-standard TRL staging system: fundamental mechanistic studies (structure-activity relationships, animal validation) correspond to TRL 1-3; early clinical exploration (phase I/II small-sample trials) corresponds to TRL 4-6; and approved clinical applications (e.g., LAG-3 inhibitors, autologous TIL therapy) correspond to TRL 7-9. Natural polysaccharide adjuvants as a class are currently positioned at TRL 3-5, fully consistent with the extant preclinical and early clinical evidence base (86). Xu et al. addressed a core bottleneck limiting β-glucan-based immunotherapeutics from progressing to higher TRL stages: the absence of highly sensitive quantitative methods for polysaccharides, attributable to their complicated chemical structures and susceptibility to endogenous interference. By establishing a UHPLC-MS/MS strategy based on a structure-specific trisaccharide fragment, they achieved absolute quantification of the Antarctic β-1,3/1,6-glucan BG136 across cellular uptake, preclinical, and clinical pharmacokinetic models—marking the first practical application of this strategy in complex biological matrices and providing methodological support for the successful execution of the BG136 Phase I clinical trial. Their work exemplifies that unified bioactive structural quality markers, coupled with robust quantitative characterization methods, are prerequisites for advancing β-glucan adjuvants into late-phase clinical trials (87). To move beyond subjective appraisal, **Table 4** applies the NASA-originated Technology Readiness Level (TRL) scale. This reveals that while FMT-based strategies approach clinical maturity (TRL 6-7), most novel polysaccharide structures remain confined to preclinical development (TRL 3-5), constrained primarily by manufacturing inconsistencies.

**Table 4:** Technology readiness level (TRL) assessment and developmental bottlenecks.

Adjuvant strategy	Representative example	Current TRL	Key developmental bottleneck	Recommended action
Crude Extracts​	Ganoderma lucidum extracts	**3**​ (Lab validation)	Structural heterogeneity; Batch variability	Implement ESAR framework (25)
Purified Polysaccharides​	Sulfated Astragalus PS	**4-5**​ (Animal/Early Human)	Poor PK/PD data; Low bioavailability	Nanodelivery systems (101, 102)
Glycan-Based Nanovaccines​	Neoantigen + Lycium PS	**5**​ (Preclinical efficacy)	Scalable GMP synthesis; Complex QC	AI-driven glycomics (92)
FMT + Prebiotic Priming​	FMT-LUMINate protocol	**6-7**​ (Phase II Clinical)	Regulatory hurdles; Donor screening	Standardized microbiome signatures (50)

Technology Readiness Levels (TRLs) are assigned according to the NASA-derived, immuno-oncology-adapted framework of Wang et al. (86): TRL 1–3, fundamental mechanistic validation *in vitro* and in syngeneic animal models; TRL 4–6, early-phase clinical exploration (Phase I–II, small-sample trials); TRL 7–9, late-phase clinical validation and regulatory-approved applications. The “Key Developmental Bottleneck” column is categorized into four predefined domains: (i) Structural heterogeneity, encompassing batch-to-batch variability, non-standardized extraction, and undefined structure–activity relationships; (ii) Pharmacokinetic/pharmacodynamic (PK/PD) deficits, including low oral bioavailability, lack of systemic exposure data, and unclear dose–response relationships; (iii) Translational infrastructure gaps, such as non-scalable GMP synthesis and complex quality control requirements; (iv) Regulatory and clinical hurdles, including donor screening for fecal microbiota transplantation, heterogeneous trial endpoints, and long-term safety surveillance. The “Recommended Action” column provides targeted, literature-substantiated mitigation strategies: implementation of the Extraction–Structure–Activity Relationship (ESAR) framework (25), adoption of AI-driven glycomics and atom-level machine learning models (92), deployment of lymph-node-targeted or stimuli-responsive nanodelivery systems (101, 102), and establishment of standardized microbiome signatures for patient stratification (50). All evaluations are therefore anchored to objective, peer-reviewed criteria rather than subjective author judgment.

Bold values indicate the current Technology Readiness Level (TRL) assigned to each adjuvant strategy.

### 6.2 Green manufacturing and structural characterization

Ji X et al. systematically summarized three core directions for green manufacturing of polysaccharides: low-energy green extraction (maximizing retention of native triple-helix/branched structures), targeted green modification (acetylation/sulfation for directional structural optimization), and synthetic biology construction (controlled *de novo* synthesis of specific bioactive structures). This triad addresses batch-to-batch heterogeneity at its source and provides the technological foundation for sustainable polysaccharide development (88). Xiong Y et al. reviewed frontier techniques for structural characterization of bioactive polysaccharides from traditional Chinese medicines, establishing that molecular weight distribution, glycosidic bond configuration, triple-helix conformational integrity, and characteristic group substitution degrees are core quality control metrics. They advocated for multidimensional structural profiling to replace the traditional “total sugar content” singular metric (89).

### 6.3 AI-optimized polysaccharide biomanufacturing

Majeed and Iftikhar defined the industry 6.0 paradigm as a transformative shift toward intelligent, sustainable manufacturing ecosystems driven by edge computing, artificial intelligence (AI), and the Internet of Things (IoT) (90). Their framework emphasizes that IoT-driven infrastructures, integrating scalable cloud platforms with localized edge-AI processing, maximize production efficiency while lowering latency and energy consumption. This architecture supports real-time anomaly detection, predictive maintenance, and autonomous decision-making. To safeguard industrial data integrity within networked environments, they advocate for blockchain-based security frameworks coupled with quantum encryption. Furthermore, they highlight the critical role of standardized communication protocols—such as MQTT and CoAP—facilitated by interoperable middleware, to harmonize disparate IoT ecosystems. Collectively, these technological advancements optimize energy utilization, drastically curtail resource consumption, and underpin a circular bioeconomy, thereby positioning Industry 6.0 as the conceptual cornerstone for AI-optimized polysaccharide biomanufacturing.

### 6.4 From QSAR to atom-level machine learning

Li Z et al. pioneered the first QSAR model for polysaccharide antioxidant activity, comparing multiple linear regression (MLR), support vector machine (SVM), and artificial neural network (ANN) algorithms. The ANN model achieved a training set R = 0.96 and test set R = 0.933, significantly outperforming conventional statistical tools. Arabinose (Ara) and galacturonic acid (GalA) were identified as the most critical structural determinants of DPPH scavenging activity, laying the methodological foundation for quantitative prediction of polysaccharide structure-activity relationships (91). Carpenter EJ et al. developed MCNet, an atom-level machine learning model that achieves high-precision prediction of interactions between glycan-binding proteins (GBPs) and glycans, breaking through the limitation of conventional monosaccharide composition-based ML models that cannot extrapolate to mirror glycan isomers. MCNet successfully predicted the binding mode of L-glucose to specific fucosylated GBPs on glycan arrays and lectin arrays, providing a novel tool for precise prediction of polysaccharide-receptor interactions (92). However, current training datasets of this model are limited to narrow glycan libraries, lacking sufficient samples of complex plant and marine polysaccharides, which restricts its universal predictive performance in translational research.

### 6.5 Synthetic biology construction of medical polysaccharides

Deng et al. reconstructed a heparosan biosynthetic pathway in engineered *Escherichia coli* K5, introducing the non-natural monosaccharide N-trifluoroacetylglucosamine (GlcNTFA) into the bacterial capsule polysaccharide to enable efficient N-site sulfation—a longstanding bottleneck in microbial heparin synthesis. By deploying the PROSS-FRISM platform, they engineered a human N-sulfotransferase mutant (NST-M8) with 11.32-fold enhanced stability and 2.53-fold increased activity, then executed a multienzyme cascade reaction to install N-sulfation, C5-epimerization, and O-sulfation (via 2-OST, 6-OST, and 3-OST) in a programmable manner. This cellular-system-based semisynthetic strategy produced structurally homogeneous anticoagulant heparin polysaccharides with anti-FXa activity of 246.09 IU/mg and anti-FIIa activity of 48.62 IU/mg, providing an animal-free, customizable solution for structurally defined medical polysaccharide production (93). Maeda K et al. achieved the first heterologous reconstruction of a complex sulfated polysaccharide biosynthetic gene cluster in cyanobacteria: transferring the synechan biosynthesis and regulatory gene cluster from *Synechocystis* PCC6803 into *Synechococcus elongatus* PCC 7942, which natively produces no sulfated polysaccharides. The engineered strain synthesized a sulfated polysaccharide composed of glucose, galactose, and mannose with sulfation content comparable to native synechan, providing proof-of-concept for cyanobacterial cell factories in producing medical sulfated polysaccharides (94).

### 6.6 Large-scale N-glycomics and AutoML diagnostics

Fu B et al. mapped the dynamic serum N-glycome across the full progression continuum from health → chronic hepatitis B → liver cirrhosis → hepatocellular carcinoma (HCC) in a 1074-participant multicenter Chinese cohort. They identified that highly branched, fucosylated, and polysialylated tri-/tetra-antennary complex N-glycans were specifically upregulated in HCC. TCGA transcriptomic validation confirmed MGAT5 (GnT-V) upregulation >3-fold, FUT8 (core fucosylation) upregulation 1.7-fold, and FUT3 upregulation 4-fold in HCC. An AutoML diagnostic model built on 26 N-glycan features achieved an AUC of 0.84-0.93, significantly outperforming the traditional marker AFP (95). Xie Y et al. extended data-independent acquisition (DIA) mode to released glycan analysis, developing the GlycanDIA workflow and its companion open-source search engine GlycanDIAFinder. In N-glycan analysis, identification throughput exceeded traditional DDA by 35% (over 270 glycans), technical replicate quantitative CV <5%, detection sensitivity reached femtomole levels, and the method effectively distinguished α2,3-/α2,6-sialic acid linkage isomers and low-abundance acetylation modifications, providing a high-throughput tool for fine structural resolution of polysaccharides/glycoproteins (96).

### 6.7 AI Multi-omics and resistance mechanisms

Cai et al. integrated 821 fecal metagenomes from 12 independent datasets spanning diverse demographic regions and cancer types, constructing random forest models that achieved an average AUC of 0.810 for predicting ICI response across multicenter cohorts—outperforming single-omics taxonomic classifiers. Pathway analyses revealed that quorum sensing, ABC transporters, flagellar assembly, and amino acid biosynthesis pathways were consistently enriched between responders and non-responders across all 12 datasets, with *luxS*, *manA*, *fliC*, and *trpB* exhibiting concordant changes. Follow-up microbiota transplant experiments demonstrated that inter-species signaling mediated by *luxS*-synthesized autoinducer-2 (AI-2) molecules acts on overall community function to promote the colonization of *Akkermansia muciniphila*, which is associated with superior ICI responses—establishing that gut microbiota functional pathways, rather than taxonomic profiles alone, are core predictors of ICI efficacy (97). Lauss M et al. performed multi-oms analysis on paired samples from 44 patients with metastatic melanoma receiving ICI therapy, discovering that ~25% of progressive lesions harbored acquired genetic alterations (e.g., JAK1/2, B2M mutations), while 75% of resistance was driven by non-genetic mechanisms mediated by the tumor microenvironment (including T cell exhaustion, myeloid-derived suppressor cell infiltration, and chemokine expression downregulation). This provides a theoretical basis for intervening in non-genetic resistance—such as TME remodeling by polysaccharide adjuvants (98). Derosa L et al. conducted a prospective shotgun metagenomics-based microbiome analysis in a cohort of 338 patients with advanced NSCLC receiving first- or second-line ICI therapy, validating the predictive value of baseline stool *Akkermansia muciniphila* (Akk). In multivariate analysis, baseline stool Akk was significantly associated with increased objective response rate (ORR) and overall survival (OS), independent of PD-L1 expression, antibiotic use, and performance status. Notably, antibiotic use (accounting for 20% of the cohort) coincided with Akk relative abundance >4.8% together with *Clostridium*-dominant composition, both of which correlated with ICI resistance—establishing that Akk is not simply “the higher the better,” but rather operates within a defined therapeutic window (99).

### 6.8 Nanodelivery system optimization

Bao et al. identified NLBPE1, a homogeneous arabinogalactan-rich polysaccharide isolated from *Lycium barbarum*, featuring an atypical arabinose-bridged structure … Moreover, combination with anti-PD-1 immune checkpoint blockade yields synergistic antitumor responses in a melanoma model—establishing NLBPE1 as a structurally and mechanistically distinct polysaccharide adjuvant with translational potential for next-generation cancer nanovaccine strategies (19). Complementary to this natural polysaccharide approach, Cui et al. employed an in silico-guided strategy to discover a benzoylated inulin derivative (InBz) as a novel TLR4-agonistic adjuvant for nanovaccine platforms (100). InBz-based nanoparticle vaccines encapsulating model antigen OVA effectively activated TLR4 signaling, facilitated dendritic cell maturation, elicited robust antigen-specific cytotoxic T lymphocyte responses, and achieved significant tumor suppression in murine models—providing a computational design paradigm for next-generation polysaccharide-derived nanovaccine adjuvants (100). He A et al. constructed a lymph node-targeted *Astragalus* polysaccharide nanovaccine, achieving passive lymph node targeting via mannose receptor ligand surface modification, significantly promoting dendritic cell maturation and antigen presentation within lymph nodes. In melanoma models, tumor suppression with combined anti-PD-1 reached 68%, far exceeding free polysaccharide (32%) and monotherapy (28%) arms (101). Gu et al. encapsulated the natural polysaccharide immunopotentiator *Angelica sinensis* polysaccharide (ASP) into polyethylenimine (PEI)-coated PLGA nanoparticles, constructing a positively charged nanoparticle delivery system (ASP-PLGA-PEI) that targets and activates antigen-presenting cells. The cationic nanoparticles significantly activated macrophages, promoting the expression of MHC-II and CD86 costimulatory molecules and the production of IL-1β and IL-12p70 cytokines. When antigens were adsorbed onto the surface of ASP-PLGA-PEI nanoparticles, antigen uptake by macrophages was markedly enhanced. *In vivo*, mice immunized with PCV2 antigen adsorbed onto ASP-PLGA-PEI nanoparticles exhibited significantly enhanced PCV2-specific IgG responses and elevated cytokine levels, inducing a mixed Th1/Th2 immune response with Th1 bias compared to control groups. These findings demonstrate that nanocapsulation effectively mitigates the *in vivo* delivery deficits of natural polysaccharides, establishing PLGA-based nanoparticle encapsulation as an effective vaccine delivery and adjuvant system (102).

### 6.9 Triple-combination therapeutic strategies

Wu et al. evaluated β-glucan combined with anti-PD-L1 blockade in subcutaneous and orthotopic pancreatic tumor models with incomplete microwave ablation (MWA), demonstrating that β-glucan elevated MHCII+ dendritic cells and that combination therapy increased the CD8+/Treg ratio—suggesting enhanced anti-tumor immunity. This immunomodulatory effect translated into significantly greater efficacy in suppressing residual tumor growth and prolonging overall survival compared to monotherapy arms. β-glucan administration reversed the immunosuppressive tumor microenvironment in pancreatic cancer following MWA ablation, thereby enhancing the anti-PD-L1-associated antitumor responses—establishing β-glucan as a potent adjuvant that mitigates the intrinsic resistance of ablation-induced immunosuppressive TME to immune checkpoint blockade (103). In parallel, Lan J et al. constructed an APS-based paclitaxel nanoplatform (APS-PTX NPs) in which APS acted concurrently as carrier, GLUT-targeting ligand, and immunomodulator. In triple-negative breast cancer models, APS-PTX NPs suppressed tumors by 92.28%, surpassing free paclitaxel (45%) and liposomal paclitaxel (68%), and additionally induced immunogenic cell death, lowered PD-L1 expression, and blunted paclitaxel-induced myelosuppression (58). A complementary example outside the Astragalus family comes from Kim et al., who used chitosan-coated nanostructured lipid carriers to co-deliver paclitaxel and PD-L1 siRNA (P-NLC-Chi-siRNA); the system reversed P-gp-mediated efflux in MCF-7/ADR cells, kept siRNA intact in serum for 24 h, and regressed xenografts in nude mice, while PD-L1 mRNA knockdown recruited CD4^+^/CD8^+^ T cells into tumors (104).

### 6.10 Co-delivery and *in situ* ablation

Song S et al. engineered all-in-one glycol chitosan nanoparticles (CNPs) that co-deliver doxorubicin (DOX) and an anti-PD-L1 peptide (PP). The PP-conjugated CNPs promote multivalent binding to PD-L1 on tumor cell surfaces, inducing lysosomal PD-L1 degradation—overcoming the recycling limitation of conventional anti-PD-L1 antibodies. In CT26 colon cancer models, DOX-PP-CNPs simultaneously triggered immunogenic cell death and PD-L1 blockade, achieving a 60% complete response rate via robust antitumor immune activation (105). This glycan-based co-delivery paradigm complements the HA-coated triple-drug nanosystem (HTCP-Au/shPD-L1/DOX) developed by Ma C et al. (57), collectively demonstrating that polysaccharide-derived nanocarriers can integrate chemotherapy, immune checkpoint blockade, and immunogenic cell death into a single programmable platform.

### 6.11 Safety boundaries (core caution)

Singh V et al. made a seminal discovery: in dysbiotic mouse models, excessive intake of fermentable soluble fiber (inulin) can induce cholestatic liver cancer (HCC). This process is strictly microbiota-dependent—occurring exclusively in non-intervened dysbiotic mice but absent in germ-free or antibiotic-treated mice. The mechanism involves excessive short-chain fatty acid accumulation leading to bile acid metabolic disorders, hepatic neutrophil infiltration, and cell death. Inhibiting fermentation or depleting SCFA-producing bacteria completely prevented HCC onset (106).This finding establishes a critical safety principle for polysaccharide/fiber adjuvants: “more is not always better.”​ Dose-dependent risk profiles and microbiome baseline assessments are essential for clinical dosing optimization and safety monitoring, balancing efficacy narratives with rigorous risk acknowledgment.

### 6.12 Multi-mechanistic drivers of cold tumors

The conceptual framework of “reprogramming the tumor microenvironment to convert cold into hot tumors,” as articulated by Yuan et al. (107), has crystallized into a central paradigm in modern immuno-oncology. Extending this perspective, Huang, et al. (108) recently provided a comprehensive, multi-omics-driven dissection of the multifactorial nature of cold tumors. They delineated a core signaling network—encompassing WNT/β-catenin hyperactivation, TGF-β-mediated immunosuppression, metabolic dysregulation (e.g., lactate accumulation), aberrant immune checkpoint expression, and vascular anomalies—that collectively orchestrates immune exclusion. Crucially, the sheer complexity of this network precludes durable clinical responses from single-agent interventions, including polysaccharide adjuvants administered as monotherapies (108). Instead, multidimensional combinatorial regimens are imperative. Beyond conventional immune checkpoint inhibitors, these strategies necessitate the integration of TGF-β antagonism, chemokine-directed therapies, oncolytic virotherapy, vascular normalization, and metabolic interventions (e.g., LDHA or GLS inhibition) (108). Within this therapeutic ecosystem, polysaccharide adjuvants—capable of dendritic cell training, myeloid compartment reprogramming, and synergistic potentiation of ICI efficacy (19, 87, 103)—constitute a critical immunomodulatory axis. While the heuristic of “cold-to-hot transition” (107) aptly describes this ambition, Huang, et al. ​ (108) caution that complete phenotypic conversion remains an aspirational endpoint, constrained by translational hurdles such as data heterogeneity and on-target toxicities. This nuanced perspective tempers expectations regarding the standalone potency of polysaccharide adjuvants, rightly situating them as indispensable components within the broader combinatorial armamentarium required to dismantle the immunosuppressive circuitry of refractory tumors.

## 7 Conclusion

This review synthesizes the translational landscape of polysaccharides as ICI potentiators, bridging structural biology with clinical pharmacology. We delineate the critical structure-activity relationships—specifically, the indispensable triple-helix conformation in fungal β-glucans and the immunoenhancing roles of acetylation and sulfation—that govern receptor engagement (e.g., Dectin-1, TLR4) and downstream signaling. Mechanistically, polysaccharides reprogram the tumor microenvironment by polarizing tumor-associated macrophages toward an M1 phenotype, reinvigorating exhausted CD8+ T cells, and remodeling gut microbiota to elevate short-chain fatty acid production. Crucially, clinical data demonstrate that combining β-glucans with ICIs or local ablative therapies can extend median overall survival to 32.5 months in pancreatic cancer and achieve objective response rates of 21.7% in advanced NSCLC, underscoring their therapeutic potential. We also map the “adjuvant efficacy spectrum”, positioning polysaccharides as a high-tolerance alternative to cytotoxic combinations. However, significant hurdles persist, including batch-to-batch structural heterogeneity, suboptimal bioavailability, and the nuanced risk of microbiota-dependent hepatocarcinogenesis under excessive dosing. Looking forward, we propose a roadmap leveraging Industry 6.0 principles: integrating AI-driven glycan modeling, synthetic biology for standardized production, and lymph-node-targeted nanodelivery systems. By harmonizing these innovations with precision biomarker strategies, such as *Akkermansia muciniphila* profiling, polysaccharides can evolve from empirical supplements into rationally designed, first-in-class adjuvants for next-generation cancer immunotherapy.

## Glossary

AE: Adverse Event
CAR-T: Chimeric Antigen Receptor T-Cell
CD44: Cluster of Differentiation 44
CD8+ T cell: CD8-Positive Cytotoxic T Lymphocyte
DCR: Disease Control Rate
Dectin-1: Dendritic Cell-Associated C-Type Lectin 1
EMT: Epithelial-Mesenchymal Transition
EPR: Enhanced Permeability and Retention
ESAR: Extraction-Structure-Activity Relationship
FMT: Fecal Microbiota Transplantation
Gal-3: Galectin-3
gMDSC: Granulocytic Myeloid-Derived Suppressor Cell
HA: Hyaluronic Acid
HG: Homogalacturonan
ICI: Immune Checkpoint Inhibitor
IDO: Indoleamine 2,3-Dioxygenase
IL: Interleukin
kLa: Oxygen Mass Transfer Coefficient
LAG3: Lymphocyte Activation Gene 3
MAPK: Mitogen-Activated Protein Kinase
mAb: Monoclonal Antibody
MMP-9: Matrix Metalloproteinase 9
MSS: Microsatellite-Stable
MyD88: Myeloid Differentiation Primary Response 88
NF-κB: Nuclear Factor-Kappa B
NRF2: Nuclear Factor Erythroid 2-Related Factor 2
NSCLC: Non-Small Cell Lung Cancer
ORR: Objective Response Rate
OS: Overall Survival
PD-1: Programmed Cell Death Protein 1
PD-L1: Programmed Cell Death Ligand 1
PFS: Progression-Free Survival
PK: Pharmacokinetic
PRR: Pattern Recognition Receptor
RECIST: Response Evaluation Criteria in Solid Tumors
RG I: Rhamnogalacturonan I
SCFA: Short-Chain Fatty Acid
STAT3: Signal Transducer and Activator of Transcription 3
Syk: Spleen Tyrosine Kinase
TAM: Tumor-Associated Macrophage
TGF-β: Transforming Growth Factor-Beta
TLR: Toll-Like Receptor
Tim-3: T-Cell Immunoglobulin and Mucin Domain-Containing Protein 3
TME: Tumor Microenvironment
Treg: Regulatory T Cell.
